# Cost-effectiveness of immune checkpoint inhibitors in treating metastatic urothelial cancer

**DOI:** 10.3389/fphar.2024.1281654

**Published:** 2024-03-26

**Authors:** Li-Yu Yang, Jian-Ri Li, Chuan-Shu Chen, Chen-Li Cheng, Sheng-Chun Hung, Kun-Yuan Chiu, Cheng-Kuang Yang, Chiann-Yi Hsu, Shian-Shiang Wang

**Affiliations:** ^1^ Department of Urology, Taichung Veterans General Hospital, Taichung, Taiwan; ^2^ Division of Surgical Intensive Care Unit, Department of Intensive Care, Taichung Veterans General Hospital, Taichung, Taiwan; ^3^ Institute of Medicine, Chung Shan Medical University, Taichung, Taiwan; ^4^ Department of Medicine and Nursing, Hungkuang University, Taichung, Taiwan; ^5^ Institute of Biomedical Science, National Chung Hsing University, Taichung, Taiwan; ^6^ Department of Applied Chemistry, National Chi Nan University, Nantou, Taiwan; ^7^ Department of Medical Research, Taichung Veterans General Hospital, Taichung, Taiwan

**Keywords:** immune checkpoint Inhibitors, urothelial carcinoma, cost-effectiveness, immunotherapy, bladder cancer

## Abstract

**Objectives:** Immune checkpoint inhibitor (ICI) is an important treatment option for metastatic urothelial carcinoma (mUC) patients. A lot of clinical evidence proved the survival benefits of ICI, but cost-effectiveness of the treatment remains unclear. This study evaluates the cost-effectiveness of the ICIs treatment in different sequences among mUC patients.

**Methods:** We retrospectively analyzed mUC patients who had been treated at our hospital between January 2016 and December 2020. These patients received chemotherapy with or without ICI treatment (Pembrolizumab, Atezolizumab, Nivolumab, Durvalumab, or Avelumab). The patients were divided into three different groups: receiving chemotherapy alone, receiving a combination of first-line ICI and chemotherapy (ICI combination therapy), and receiving chemotherapy as the first-line treatment followed by second-line ICI therapy (Subsequent ICI therapy). The primary endpoint was cost per life day, while lifetime medical costs and overall survival were also evaluated.

**Results:** The 74 enrolled patients had a median age of 67.0 years, with 62.2% being male. Of these patients, 23 had received chemotherapy only, while the remaining patients had received combined therapy with ICI in either first-line or as subsequent agents (37 patients had ever received atezolizumab, 18 pembrolizumab, 1 Durvalumab, 1 Nivolumab, and 1 Avelumab separately.). Fifty-five patients (74.3%, 55/74) received cisplatin amongst all the patients who underwent chemotherapy. Median overall survival was 27.5 months (95% CI, 5.2–49.9) in the first-line ICI combination therapy group, and 8.9 months (95% CI, 7.1–10.8) in the chemotherapy only. Median overall survival for the subsequent ICI therapy group was not reached. The median lifetime cost after metastatic UC diagnosis was USD 31,221. The subsequent ICI therapy group had significantly higher costs when compared with the ICI combination therapy group (155.8 USD per day, [IQR 99.0 to 220.5] v 97.8 USD per day, [IQR 60.8 to 159.19], *p* = 0.026). Higher insurance reimbursement expenses for the subsequent ICI therapy group were observed when compared with the ICI combination therapy group.

**Conclusion:** Our real-world data suggests that first line use of ICI combined with chemotherapy demonstrates better cost-effectiveness and similar survival outcomes for mUC patients, when compared with subsequent ICI therapy after chemotherapy.

## 1 Introduction

Platinum-based chemotherapy has been the standard first-line systemic treatment for metastatic urothelial carcinoma (mUC) for decades, with the response rate ranging from 43.6% to 55.5%, and an overall survival (OS) period of 12.7–15.9 months, based upon different regimens (von der Maase et al., 2005; Bellmunt et al., 2012). However, approximately half of all mUC patients is ineligible for cisplatin treatment due to poor performance status, comorbidities, or impaired renal function (Bamias et al., 2018). For these patients, carboplatin-based regimens are alternative options in platinum eligible patients, resulting in a median overall survival of 9–10 months (Bukhari et al., 2018).

During the past 5 years, inhibitors of programmed cell death-1 (PD-1)/programmed death-ligand 1 (PD-L1), such as pembrolizumab, nivolumab, atezolizumab, avelumab and durvalumab have been considered as a standard second-line treatment for patients with disease progression after undergoing treatment with platinum-based chemotherapy, while also being a first-line option for cisplatin-ineligible patients who have PD-L1 positive tumors (Balar et al., 2017a; Apolo et al., 2017; Bellmunt et al., 2017; Powles et al., 2017; Sharma et al., 2017; Patel et al., 2018).

Based upon the clinical benefits which both chemotherapy and immune checkpoint inhibitors (ICIs) demonstrating in mUC treatment, efforts are currently underway to evaluate a combined approach in a frontline setting. Previous phase III studies involving both KEYNOTE-361 and IMvigor 130 showed no differences in overall survival (OS) improvement among patients receiving pembrolizumab or atezolizumab in combination with chemotherapy when compared with chemotherapy alone (Galsky et al., 2020; Powles et al., 2021; Luo et al., 2022). However, the phase 3, randomized controlled trial, CheckMate 901 recently reported that nivolumab plus gemcitabine–cisplatin significantly improved OS and progression-free survival in previously untreated advanced UC patients (van der Heijden et al., 2023). This breakthrough suggests that the nivolumab and chemotherapy combination could be a new option for these patients.

The adverse events of chemotherapy including nausea, vomit or fatigue might discourage the patients to receive treatment and the chemotherapy related leukopenia, fever or dehydration may hospitalize the patients. Although the side effects of ICI are minor than the chemotherapy, immune related adverse events such as skin rash, colitis, pneumonitis, or hypothyroidism are still needed to be concerned. In addition, the treatment related complications may increase the emergency room visits or hospitalization and then cause the higher medical expenses.

Although the sequential or combination use of chemotherapy and ICI might provide clinical benefits for advanced UC patients, the financial burden of treatment is also an important issue for the patients and the insurance system. It is necessary to analyze the cost-effectiveness of treatment to find the balance of clinical outcomes and medical cost. However, most cost-effectiveness analyses predominantly focus on medication costs alone. It is essential to encompass the overall expenses in healthcare to represent the real-world economic burdens more accurately. Through this retrospective patient series study, we evaluated both overall survival and total medical care expenses in metastatic UC patients to provide clinical evidence regarding the cost-effectiveness of ICI treatment in different sequences.

## 2 Materials and methods

### 2.1 Patients

This retrospective study was conducted through both chart review and analysis of hospital databases, including patients who either underwent all self-payment treatment or were receiving National health insurance (NHI) reimbursements. This study was approved by the Institute Review Board of Taichung Veterans General Hospital (Number CE21353B). Between 1 January 2016, and 31 December 2020, we recruited metastatic UC patients who had received chemotherapy with or without ICI treatments. ICI drugs included two PD-1 inhibitors (pembrolizumab and nivolumab), and three PD-L1 inhibitors (atezolizumab, durvalumab and avelumab). Patients were excluded if they had been diagnosed with another cancer within the 5 years prior to UC diagnosis or had received any research funding.

### 2.2 Study assessment

Our object is to evaluate the lifetime economic burden of the metastatic UC patients. Starting from the time when the UC patient was diagnosed with metastasis, the total medical expenses including inpatient fees, emergency room visit costs, and outpatient fees were calculated. The primary endpoint is the cost per life day (the ratio of the total lifetime cost to OS with treatment per patient). We also evaluated lifetime cost, OS, and tumor response to chemotherapy and ICI treatment. OS was calculated from the time of metastasis diagnosis to either death or the end time of this study (December 2020). Baseline patient characteristics included the following: age, gender, primary tumor site, survival status, treatment model, chemotherapy response, ICI response, and comorbidities such as hypertension, diabetes mellitus, chronic kidney disease (CKD) and end-stage renal disease (ESRD). We divided the patients into different groups based upon the following conditions: (a) receiving chemotherapy alone; (b) receiving a combination of first-line ICI and chemotherapy (ICI combination therapy); and (c) receiving chemotherapy as the first-line treatment followed by second-line ICI therapy (Subsequent ICI therapy). Chemotherapy and ICI responses were recorded according to the Response Evaluation Criteria in Solid Tumors (RECIST version 1.1). Objective response rate was the proportion of patients who achieved either a complete response (CR) or a partial response (PR). The periods of radiographic evaluation varied between 4 weeks and 6 months, depending on the clinical condition of each individual patient.

### 2.3 Statistical analysis

Differences in continuous variables were assessed using the Mann-Whitney U and Fisher’s exact tests. The Chi-square test was used for categorical variables. The OS curves were plotted according to the Kaplan–Meier method. Cumulative lifetime costs were survival-adjusted from the Kaplan-Meier curves, according to either the time to death or end time of the study. All analyses were performed using SAS software version 9.2 (SAS Institute, Cary, NC, USA).

## 3 Results

During the study period, a total of 81 patients in our database received chemotherapy with or without ICI for the treatment of metastatic UC. Of them, 7 who had received research funding were excluded from our study.

**FIGURE 1:**
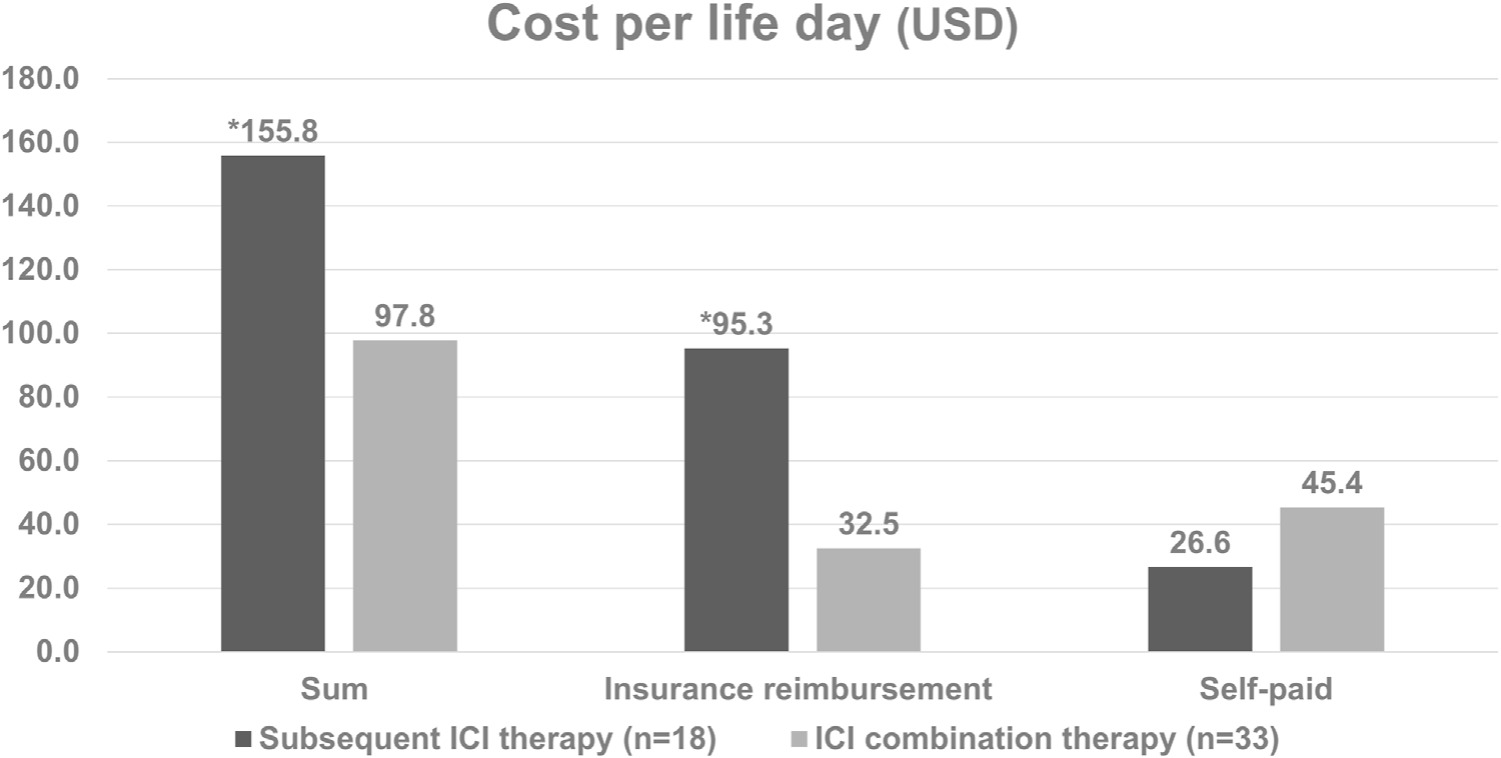
Cost per life day based on the type of treatment in patients with metastatic diseases. *The subsequent ICI therapy group had significantly higher daily costs per life day compared to the ICI combination therapy group (*p* = 0.026). Additionally, insurance reimbursement expenses were higher for subsequent ICI therapy (*p* = 0.011).

The baseline characteristics of the patients are listed in Table 1. Of the 74 enrolled patients, the median age was 67.0 years (range 58.3–73.6), with 62.2% being male. Twenty-one patients (28.4%) had upper urinary tract urothelial carcinoma (UTUC), 29 (39.2%) had urothelial carcinoma of the urinary bladder (UBUC) and 24 (32.4%) had concomitant UTUC and UBUC. The common comorbidities seen in the UC patients were hypertension (59.5%), diabetes (37.8%) and CKD (44.6%). Regarding treatments, 33 patients enrolled in the ICI combination group received first line ICI plus chemotherapy, 18 received subsequent ICI therapy after first line chemotherapy while the other 23 received chemotherapy alone. Amongst all patients, 73 (98.6%, 73/74) had received platinum-based chemotherapy in our hospital, 74.3% (55/74) received cisplatin, and one received paclitaxel as their chemotherapy dominant regimen. Among these patients, 37 had received Atezolizumab, 18 Pembrolizumab, 1 Durvalumab, 1 Nivolumab, and 1 Avelumab. These immunotherapy treatments were divided into the ICI combination therapy and subsequent ICI therapy groups. In addition to platinum base chemotherapy, the combination therapy group patients received different ICIs including atezolizumab in 30 patients, pembrolizumab in 7 patients, nivolumab in 1 patient, and avelumab in 1 patient as part of the combination therapy. The subsequent ICI therapy group patients received atezolizumab in 7 patients, pembrolizumab in 11 patients, and durvalumab in 1 patient (Table 1). The median lifetime cost after metastatic UC diagnosis was USD 31,221. Hospitalization expenses were the primary cost driver of lifetime costs, comprising approximately 75.0% of all costs, followed by outpatient costs (23.4%). Among the patients whose responses could be evaluated, a total of twenty-three individuals (69.7%, 23 out of 33) achieved either complete response (CR) or partial response (PR) in the ICI combination therapy group. In the subsequent ICI therapy group, three patients (16.7%, 3 out of 18) responded with CR/PR to chemotherapy, while eight patients (44.4%, 8 out of 18) experienced CR/PR after receiving subsequent ICI therapy (Table 2). One patient lacked the necessary available information regarding their chemotherapy response, and two patients had incomplete records pertaining to ICI response data in the subsequent ICI therapy group. The subsequent ICI therapy group experienced a significantly higher cost per life day when compared with the ICI combination therapy group (USD 155.8 per day, [IQR 99.0 to 220.5] vs. USD 97.8 per day, [IQR 60.8 to 159.2], *p* = 0.026). Higher insurance reimbursement expenses were observed for the subsequent ICI therapy when compared with the ICI combination therapy (USD 95.3 per day, [IQR 35.2 to 183.5] vs. USD 32.5 per day, [IQR 24.5 to 65.2], *p* = 0.011). (Figure 1).

**TABLE 1:** Baseline patient characteristics.

	ICI combination therapy (n = 33)		Subsequent ICI therapy (n = 18)		Chemotherapy alone (n = 23)		*p*-value
Age	66.4	(51.8–87.6)	64.9	(38.9–83)	67.9	(58.4–76.8)	0.655
BMI	23	(18.4–34.5)	23.1	(18.4–29.3)	22.0	(19.3–24.6)	0.381
Gender							0.525
Female	14	42.4%	6	33.3%	8	34.8%	
Male	19	57.6%	12	66.7%	15	65.2%	
Sites							0.763
UB	14	42.4%	8	44.4%	7	30.4%	
UT	10	30.3%	7	38.9%	4	17.4%	
UB + UT	9	27.3%	3	16.7%	12	52.2%	
Comorbidity							
Hypertension	21	63.6%	10	55.6%	13	56.5%	0.805
DM	15	45.5%	8	44.4%	5	21.7%	0.159
Ischemic heart disease	10	30.3%	2	11.1%	4	17.4%	0.254
Cerebrovascular disease	4	12.1%	3	16.7%	2	8.7%	0.817
Hyperlipidemia	5	15.2%	2	11.1%	4	17.4%	0.918
COPD	4	12.1%	4	22.2%	1	4.3%	0.251
Peripheral vascular disease	1	3.0%	1	5.6%	2	8.7%	0.810
Chronic kidney disease	11	33.3%	8	44.4%	14	60.9%	0.125
Gout	3	9.1%	2	11.1%	2	8.7%	1.000
Drug							
Durvalumab	0	0%	1	5.6%			
Pembrolizumab	7	21.2%	11	61.1%			
Atezolizumab	30	90.9%	7	38.9%			
Nivolumab	1	3.0%	0	0%			
Avelumab	1	3.0%	0	0%			
Cisplatin	24	72.7%	14	77.8%	17	73.9%)	0.924
Gemcitabine	32	97.0%	16	88.9%	21	91.3%)	0.509
Carboplatin	16	48.5%	10	55.6%	6	26.1%)	0.120
Methotrexate	5	15.2%	8	44.4%	4	17.4%)	0.044*
Paclitaxel	1	3.0%	4	22.2%	5	21.7%)	0.045*

Cox regression. *p < 0.05, **p < 0.01.

Data are median (range) or n (%). BMI, body mass index; UB, urinary bladder; UT, upper urinary tract; DM, diabetes mellitus; COPD, chronic obstructive pulmonary disease.

**TABLE 2:** Clinical outcomes.

	ICI combination therapy (n = 33)		Subsequent ICI therapy (n = 18)		*p*-value
Survival status					
Death	13	39.4%	8	44.4%	<0.001^*^
Survival (months)	27.5	5.2 to 49.9	Not reach		0.846
First-line C/T response					---
CR			1	5.6%	
PR			2	11.1%	
SD			4	22.2%	
PD			10	55.6%	
Unknown			1	5.6%	
Second-line IO response					---
CR			4	22.2%	
PR			4	22.2%	
SD			5	27.8%	
PD			3	16.7%	
Unknown			2	11.1%	
Combination response					---
CR	3	9.1%			
PR	20	60.6%			
SD	3	9.1%			
PD	7	21.2%			
Unknown	0	0%			
Costs (USD)					
Cost per life day					
Sum	97.8	(15.3–251.4)	155.8	(35.1–233.8)	0.026^*^
Insurance reimbursement	32.5	(10.6–165.3)	95.2	(8.7–223.3)	0.003^**^
Self-paid	45.4	(1.0–180.0)	26.6	(0.6–154.9)	0.098
Total cost	35310.4	(4217.2–191980.9)	60787.7	(17706.1–152825.9)	0.005^**^
Inpatient fee	31505.4	(1005.7–185512.6)	25747.7	(3720.2–138717.9)	0.708
Emergency room visit cost	239.5	(0–2835.00)	537.8	(0–2204.1)	0.439
Outpatient fee	2080.0	(542.8–390860.0)	6784.9	(1482.1–148447.3)	0.003^**^

Cox regression. *p < 0.05, **p < 0.01.

Data are median (range) or n (%). CR, complete response; PD, progression disease; PR, partial response; SD, stable disease; USD, united states dollars.

**FIGURE 2:**
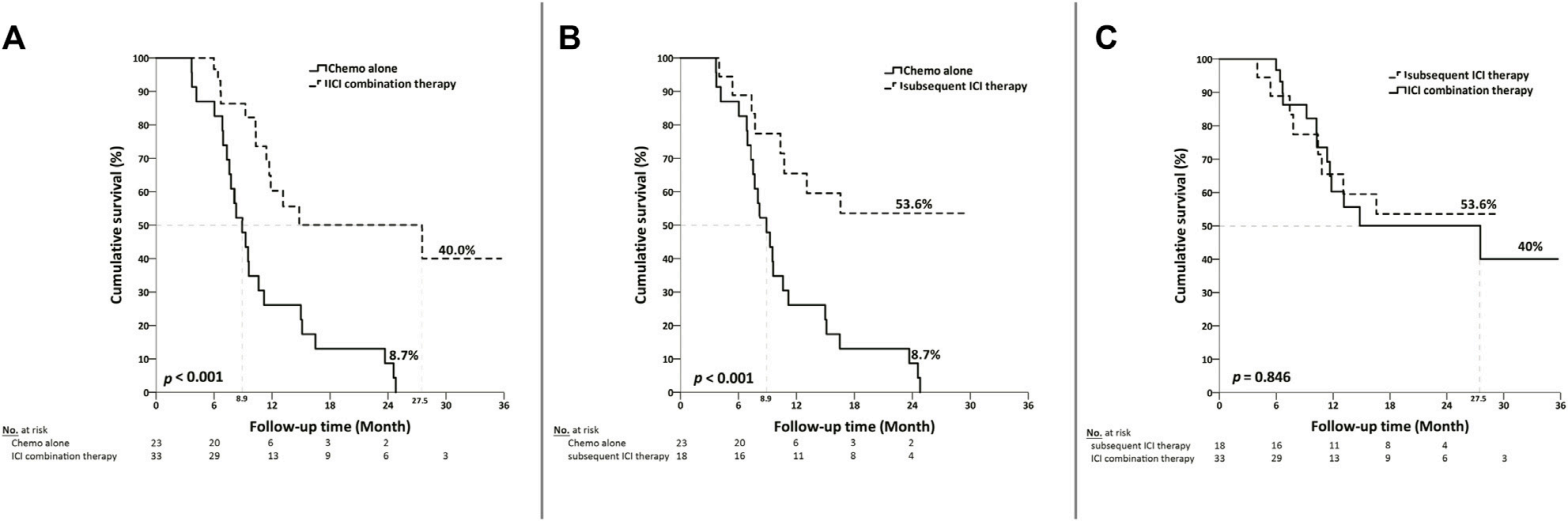
Kaplan-Meier curves depicting overall survival based on the type of treatment in patients with metastatic diseases. **(A)** Median overall survival was 27.5 months (95% CI, 5.2–49.9) in the ICI combination therapy group, compared with 8.9 months (95% CI, 7.1–10.8) in the chemotherapy group. **(B)** The median overall survival was not reached in the subsequent ICI therapy group, as compared with 8.9 months (95% CI, 7.1–10.8) in the chemotherapy group. **(C)** There was no statistically significant difference in median overall survival between the ICI combination therapy group and the subsequent ICI therapy group. (*p* = 0.846).

The median overall survival was 27.5 months (95% CI, 5.2–49.9) in the first-line ICI combination therapy group, 8.9 months (95% CI, 7.1–10.8) in the chemotherapy-only group, with the subsequent ICI therapy group not reaching the median overall survival point. Neither the ICI combination therapy nor subsequent ICI therapy group was associated with a significantly longer overall survival than the chemotherapy alone group (Figures 2A, B). There was no statistically significant difference in median overall survival between the ICI combination therapy group and the subsequent ICI therapy group. (*p* = 0.846) (Figure 2C).

## 4 Discussion

We performed this study to evaluate the cost-effectiveness of early or later use of ICI in metastatic UC patients. The results showed the combination use of ICI and chemotherapy in the first line setting spent less expenses than the sequential use of chemotherapy and then the ICI treatment. The cost per life day was USD 155.8 per day for the subsequent ICI therapy group and USD 97.8 per day the ICI combination therapy group. The subsequent group is 1.59 times more expensive than the combination group spent. The total cost per patient was also higher in the subsequent ICI therapy group than the combination therapy group (35310.4 vs. 60787.7 USD per patient, *p =* 0.005) There were no statistically significance in inpatient fee and emergency room visit cost per patient between the two groups, but the subsequent ICI group had the higher outpatient fee than the combination group. (6784.9 vs. 2080.0 USD per patient, *p* = 0.003) The median overall survival was 27.5 months in the first line ICI combination therapy group which is longer than the data in the CheckMate 901 which showed median overall survival of the nivolumab plus chemotherapy was 21.7 months (van der Heijden et al., 2023). There was no statistically significant difference in median overall survival between combination and subsequent group in our analysis although the median survival was not reached in subsequent group. There was 69.7% of our combination group patients fulfilled the criteria of complete response or partial response compared with 57.6% of complete or partial response patients in CheckMate 901 (van der Heijden et al., 2023). Better clinical outcomes with longer overall survival and higher objective response rate seemed to be found in our patient groups than the patients in CheckMate 901 trial. It is worthwhile to further explore the treatment outcomes in different patient population. According to our data, early use of ICI combined with chemotherapy is more cost-effective than the subsequent ICI use policy without compromised survival outcomes.

Chemotherapy and ICI are all standard treatment options for advanced UC patients with proved overall survival and progression free survival benefits (von der Maase et al., 2005; Bellmunt et al., 2012; Apolo et al., 2017; Balar et al., 2017a; Bellmunt et al., 2017; Powles et al., 2017; Sharma et al., 2017; Patel et al., 2018; Guo et al., 2019). Although the clinical guidelines suggested the different treatment algorithms according to the clinical efficacy, the financial burden for the individual patients or the healthcare system is another important factor to affect the treatment decision making. Contieri et al. found that the cost of medications increased after introduction of ICIs for metastatic UC in European guidelines and emphasized the crucial role of cost-effective analysis (Contieri et al., 2024). The optimal ICI treatment duration among responders is still under investigated. The ongoing phase III MOIO Protocol trial was designed to compare standard ICI treatment schedule to lower dose intensity schedule in responding patients with metastatic cancer. If the non-inferiority hypothesis of this trial is validated, the alternative treatment strategy could reduce the drug toxicity and cost without compromising efficacy and then maximize the cost-effectiveness of ICI treatment (Gravis et al., 2023). Our study showed the subsequent ICI therapy group experienced a significantly higher cost per life day when compared with the ICI combination therapy group, this indicated the early use of ICI is more cost-effective than later line use.

Besides, cost-effectiveness analysis is an important reference for the National Health Insurance Administration to reimburse the treatment. Several reports from previous clinical trials revealed controversial outcomes regarding the cost-effectiveness of ICIs when compared with chemotherapy, whether it is first-line monotherapy or second-line therapy in mUC (Sarfaty et al., 2018; Criss et al., 2019; Parmar et al., 2020; Slater et al., 2020; Srivastava et al., 2020; Hale et al., 2021). Srivastava et al. suggested pembrolizumab is more cost-effective than conventional chemotherapy based on KEYNOTE-045 results (Srivastava et al., 2020). However, their assessment only considered medication costs, omitting ongoing disease management expenses, indirectly lowering the incremental cost-effectiveness ratio. In our study, we meticulously calculated overall expenses, encompassing inpatient fees, emergency room visits, and outpatient fees, aiming for a more accurate representation of real-world economic burdens. Abdalla Aly et al. reported that pembrolizumab is more cost-effective than chemotherapy as second-line treatment at a willingness-to-pay threshold of €100000 per quality-adjusted life years for advanced UC (Aly et al., 2020). There were more insurance reimbursement expenses in the subsequent ICI therapy group in this study. (95.2 vs. 32.5 USD per life day, *p* = 0.003) This was due to our national health insurance policy, which primarily provides reimbursement for ICI treatments to patients who are in the second line treatment or beyond.

Our study data were obtained between 2016 and 2020, with most ICI treatment involving atezolizumab and pembrolizumab. It is because that atezolizumab had received accelerated approval from the US Food and Drug Administration (FDA) for the treatment of patients with advanced or metastatic urothelial carcinoma in 2016, with pembrolizumab subsequently being approved in 2017 (Balar et al., 2017a; Balar et al., 2017b; Twomey and Zhang, 2021).

The first-line combination of ICIs with chemotherapy, as demonstrated in the phase 3 trials Keynote 361 and IMvigor 130 (Galsky et al., 2020; Guo and Lin, 2020; Powles et al., 2021), revealed no significant difference in OS improvement when compared with the group receiving chemotherapy alone. In this study, the median OS was significant longer in either ICI combination or ICI subsequent treatment group than the patients received chemotherapy alone and without any other subsequent treatment. (combine ICI vs. chemotherapy alone: 27.5 vs. 8.9 months, *p* < 0.001; subsequent ICI vs. chemotherapy alone: not reach vs. 8.9 months, *p* < 0.001) There was no statistically significant difference in median OS between the ICI combination therapy group (27.5 months) and the subsequent ICI therapy group (not reached) (*p* = 0.846). Treatment for the ICI combination therapy group is more cost-effective due to its lower expenses and comparable median OS when compared with the subsequent ICI therapy group.

Cisplatin-based chemotherapy significantly increases the likelihood of both overall response (Risk ratio (RR) = 1.34) and complete response (RR = 3.54) in patients with mUC compared to carboplatin-based chemotherapy (Galsky et al., 2012). In our study, the cisplatin-based chemotherapy group accounted for the majority of platinum drugs (74.3%, 55/74). This favorable prognosis may be attributed to the high response rate (69.7%, 23 out of 33) observed in the ICI combination therapy group. This may also correspond to JAVELIN bladder 100 and Li et al. presented the predictive role of first-line chemotherapy response to ICI treatment efficacy in advanced or mUC patients (Powles et al., 2020; Li et al., 2023). Chemotherapy induction during UC treatment can deplete immunosuppressant cells, in turn increasing T-cell infiltration into tumors, antigen presentation and PD-L1 expression (McDaniel et al., 2016; Tsai et al., 2019; Li et al., 2023). The ICI combination treatment responders thus results in cost-savings and increases in cost-effectiveness.

Furthermore, extending the observation period contributes to increased cost-effectiveness. Parmar et al. demonstrated that extending the trial follow-up period from 2 years to 10 years enhances cost-effectiveness (Parmar et al., 2020). A sufficiently long follow-up period reduces the uncertainty of extrapolating the survival beyond the trial observation period. Any price reduction of ICI drugs is also expected to improve the cost-effectiveness for patients.

Limitations of this study include the following: the small number of enrolled patients, retrospective and non-randomized study design, patient selection bias, and a lack of external validation. Additionally, we were only able to collect data surrounding hospital expenses, so a lack of data regarding any medical expenses from other hospitals and clinics may act as confounding factors in our study. Also, given the period of our data collection, atezolizumab and pembrolizumab accounted for the most treatment options for first combination therapy. Finally, the PD-L1 expression was only checked in some patients, we could not explore the role of PD-L1 expression in this cost-effectiveness analysis.

In conclusion, our real-world data suggests that first line use of ICI demonstrates better cost-effectiveness and similar survival outcomes for mUC patients when compared with subsequent ICI therapy. Besides the medication cost, the impact of emergency room visits and hospitalization on total lifetime costs should be take into consideration in analyzing the cost effectiveness of treatment. Nevertheless, additional large-scale studies are still required to further validate these results.

## Data Availability

The datasets presented in this study can be found in online repositories. The names of the repository/repositories and accession number(s) can be found in the article/Supplementary material.
